# Why We Sleep: The Temporal Organization of Recovery

**DOI:** 10.1371/journal.pbio.0060106

**Published:** 2008-04-29

**Authors:** Emmanuel Mignot

## Abstract

Why we sleep seems like a simple question, yet it has baffled scientists for generations. Based on recent data, Emmanuel Mignot argues that the function of sleep is essentially a resilient form of cellular recovery, organized anatomically and temporally by natural evolution, that has taken on additional functions over time.

If sleep does not serve an absolutely vital function, then it is the biggest mistake the evolutionary process has ever made,” Allan Rechtschaffen said. Studies of sleep and sleep deprivation suggest that the functions of sleep include recovery at the cellular, network, and endocrine system levels, energy conservation and ecological adaptations, and a role in learning and synaptic plasticity.

## The Necessity of Sleep in Mammals and Birds: REM and NREM Sleep

In mammals and birds, sleep is associated with specific cortical electroencephalogram (EEG) patterns (Box 1), which may be divided into rapid eye movement (REM) and non-rapid eye movement (NREM) sleep [1]. The only known exceptions include the primitive egg-laying mammals echidnae [2], where a REM/NREM mixed state has been proposed. Some birds and marine mammals also have brief REM sleep and unihemispheric (one-sided) NREM sleep [3–5]. In some marine mammals, NREM sleep rebound is also observed in the corresponding hemisphere after selective deprivation, suggesting localization of NREM sleep and its homeostasis.

**Box 1. Mammalian Sleep and Wakefulness Defined through EEG**

**NREM sleep** is separated into light sleep (slowing of the EEG, presence of sleep spindles and K-complexes) and deep slow-wave sleep. Slow waves reflect synchronization of periods of neuronal depolarization/high firing (up-phase) followed by periods of hyperpolarization (down phase) within large areas of the cortex. Slow-wave sleep intensity is often measured by EEG power in the delta frequency range.

**REM sleep** is also called paradoxical sleep. EEG is desynchronized and hippocampal theta rhythms are present. Muscle atonia and dreaming also occur. REM sleep is often separated into “tonic REM sleep,” with atonia, and “phasic REM sleep,” with bursts of rapid eye movements and muscle twitches.

Sleep is organized in **sleep cycles**, and displays some EEG variation across mammals. REM sleep follows NREM sleep, but total amounts of sleep and sleep stages are variable [19,25,118]. Similarly, the periodicity of the cycle varies from a few minutes to a few hours [1]. **Wakefulness** is characterized by EEG desynchronization and consciousness.

Sleep is regulated by **circadian** and **homeostatic** processes [20]. Circadian regulation is demonstrated by the maintenance of a 24-hour rhythm in sleep propensity, in the absence of temporal cues. Circadian regulation anticipates environmental cues, mostly light-dark changes. Homeostatic regulation is reflected by increased sleep (sleep rebound) after sleep deprivation. During NREM sleep recovery, delta power decreases exponentially with time, tracking the dissipation of the behavioral sleep debt. REM sleep is also homeostatically regulated.

The importance of sleep is illustrated by the effects of sleep deprivation in humans [6], which is difficult to sustain for more than a few days to a week [7]. Cognition becomes impaired [8] and mood labile [6], although large inter-individual differences exist [9]. Neuroendocrine changes and microsleeps, with temporary loss of consciousness, occur [6,10]. Selective REM sleep deprivation produces similar behavioral effects over a longer time frame. In this case, increased attempts to enter REM sleep are noted, suggesting the development of a REM sleep debt [11]. Total sleep deprivation alleviates depression [12,13], but the effects are rapidly reversed by sleep. In humans, sleep deprivation is most often chronic and partial, and is increasingly recognized as having deleterious effects on human health.

In rats, total sleep deprivation is lethal after two to three weeks [14]. Within days, animals become hyperphagic but lose weight, a state associated with increased heart rate and energy expenditure. Body temperature subsequently drops. Animals are then increasingly debilitated, emaciated, and develop ulcers on the tail and paws [14]. Total REM sleep deprivation produces a similar syndrome with a longer time course [14]. However, it is difficult to dissociate the effects of sleep deprivation from stress in rats, as only humans are able to accept sleep deprivation voluntarily. The effects of long-term sleep deprivation have not been documented in other species, and sleep may not be vital for survival in all circumstances. For instance, constantly flying migrating birds and, more controversially, newborn whales (and their nursing mothers) may temporarily suspend sleep altogether without deleterious effects or need for catching up [4,5,15–17]. Thus, both REM and NREM sleep appear vital and subject to homeostasis but can be suspended in rare cases for a substantial portion of life.

In contrast to circadian biology, where the selective advantage of predicting daylight and seasonal occurrence is obvious [18], sleep is dangerous in the presence of predators. Accordingly, in mammals, REM sleep amount and length are lower in ungulates versus carnivores [19]. Similarly, ecological pressures, such as the need to keep moving for migrating birds or to breathe air for diving marine mammals, may explain unihemispheric sleep and the small amount of REM sleep in some marine mammals [19]. Further, in line with increased REM sleep early in development, altricial species (born immature and requiring parental oversight after birth) have higher amounts of REM sleep at birth [19]. The fact that NREM and REM sleep are maintained in the face of strong ecological pressures further argues for a vital function for sleep. In humans (and most mammals), sleep is regulated by the circadian clock and sleep homeostasis [20,21]. Humans are awake in the morning because sleep pressure is low after a night's rest. Throughout the day, increasingly strong wake-promoting signals, partially driven by the circadian clock, counteract the mounting sleep debt, keeping subjects awake [22]. An opposite interaction occurs during the night [21]. The circadian wake/sleep signal is approximated by body temperature fluctuations under constant conditions, peaking near 9 P.M., with a low point at 4 A.M. in humans [21].

## Sleep in Other Organisms

Sleep as a behavior is universal [1,19,23–25]. And while electrophysiology in organisms without a developed cortex (for example, turtles, lizards, and fish) has yielded controversial data [19,26], a better understanding of sleep may come from the study of non-mammalian species amenable to genetic analysis, such as Drosophila [27].

To provide a framework for experimental investigations in these simpler organisms, a behavioral definition of “sleep” has been proposed [1,19,23]: rapid reversibility (as opposed to hibernation or coma), place preference/specific position, increased arousal threshold (decreased responsiveness to sensorial stimuli), homeostatic regulation (need for recovery after deprivation), and often circadian regulation [19,23]. Studies have been extended to Drosophila [28] and zebrafish [29–32], where sleep mutants have been isolated [32,33]. Further, as in mammals, sleep and memory are connected and waking experience modulates sleep in Drosophila [34,35]. In contrast to the circadian field, however, it has been difficult to find a common core genetic pathway in sleep, possibly because few mutants have been identified. Further, while organisms such as neurospora and plants show daily cycles of activity, a definition of sleep has not been extended to these species, although a recent study has revealed a sleep-like state in Caenorhabditis elegans called lethargus [36]. Common sleep pathways in these organisms have involved dopamine [37,38], cyclic AMP response element-binding protein (CREB) [39,40], voltage-dependent potassium channels [33,41], gamma-aminobutyric acid (GABA) [42], and the epidermal growth factor [43,44].

## Anatomical Models of Sleep Regulation: Localized or Distributed?

**Figure 1:**
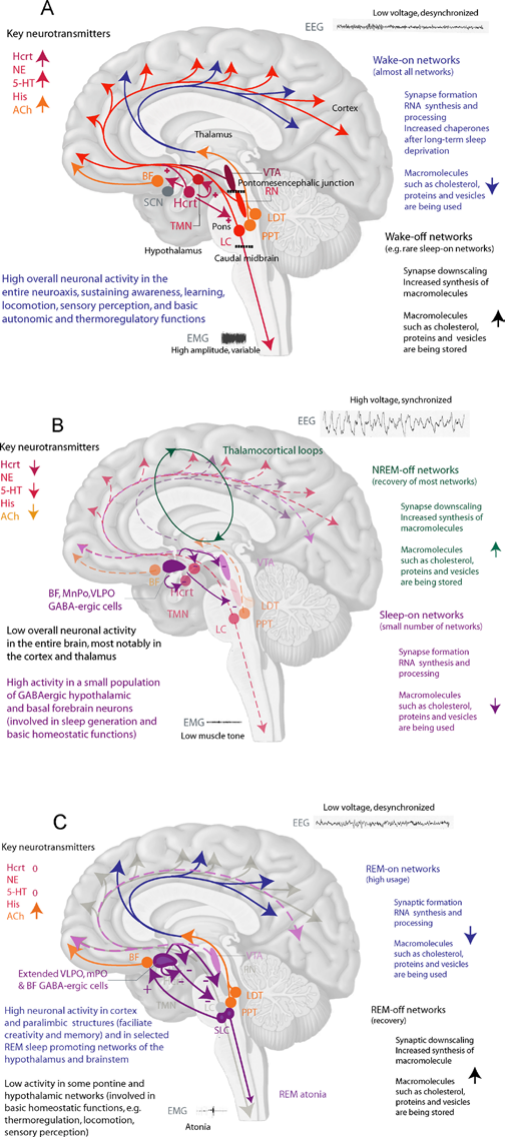
Activity of Major Neural Networks and Hypothesized Molecular Processes Occurring During Wake, NREM Sleep, and REM Sleep The different colors denote various neurotransmitter systems: red, norepinephrine (NE), histamine (His), and serotonin (5-HT); orange, acetylcholine (Ach); dark red: hypocretin (Hcrt) peptide; pink, dopamine; dark violet: GABAergic and glutamatergic NREM sleep and REM sleep-on neurons. Anatomical abbreviations: BF, basal forebrain (Ach and GABA populations); LDT, laterodorsal tegmental cholinergic (Ach) nucleus; PPT, mesopontine pedunculopontine tegmental cholinergic (Ach) nucleus; LC, adrenergic (NE) locus coeruleus; RN, serotoninergic (5-HT) raphe nuclei; TMN, histaminergic (His) tuberomammillary nucleus; VTA, dopaminergic ventral tegmental area; mPO, median preoptic hypothalamic (GABA) systems; VLPO, ventrolateral preoptic hypothalamic (GABA) systems; SLC, sublocus coeruleus area (GABA and glutamatergic cell groups); SCN, suprachiasmatic nucleus, Hcrt, hypocretin/orexin containing cell group. Upward and downward arrows represent increased/decreased activity and release for selected neurotransmitter (e.g., Ach, NE, 5-HT, His, and Hcrt) systems or in selected metabolic pathways (e.g., protein biosynthesis) during the corresponding sleep/wake state. (A) Wakefulness. During wakefulness, monoaminergic, hypocretinergic, and cholinergic systems are active and contribute to EEG desynchronization through thalamic and cortical projections. Hypocretin cells excite monoaminergic cells, and possibly cholinergic neurons (the net effect on cholinergic neurons is more difficult to estimate as most hypocretin receptors are mostly located on adjacent GABAergic cells in these regions). Muscle tone (electromyogram or EMG) is variable and high, reflecting movements. Note that dopaminergic cells of the VTA do not significantly change firing rates across sleep and wake, although pattern of firing does, contributing to higher dopaminergic release during wakefulness in the prefrontal cortex. The suprachiasmatic nucleus, the master biological clock, is located close to the optic chiasma, and receives retinal input. Time of the day information is relayed through the ventral subparaventricular zone to the dorsomedial hypothalamus, and other brain areas. Note that the SCN is not labeled in further brain diagrams. We hypothesize that during wake, activity, learning, and many metabolic processes are pushed to maximal, unsustainable levels in almost all neuronal networks to compete behaviorally at optimal times for reproduction and feeding. (B) NREM, slow-wave sleep. GABAergic cells of the basal forebrain (BF), median (mPO) and ventrolateral preoptic (VLPO) hypothalamic area are highly active during NREM sleep. mPO area GABAergic cells may also be involved in thermoregulation. VLPO and other cells inhibit monoaminergic and cholinergic cells during NREM and REM sleep. Upon cessation of sensory inputs and sleep onset, thalamocortical loops from the cortex to the thalamic reticular nucleus and relay neurons contribute to the generation of light NREM sleep. As NREM sleep deepens, slow-wave oscillations appear on the EEG. Muscle tone is low but not abolished in NREM sleep. We hypothesize that during NREM sleep, most of the brain (and most notably the cortex) as well as many peripheral organs are recovering. (C) REM sleep. During REM sleep, hypothalamic and basal forebrain sleep-on cells are active, but glutamatergic cells in the sublocus coeruleus (SLC REM-on neurons) also increase activity. These cells trigger REM atonia through caudal projections, while ventral basal forebrain projections contribute to hippocampal theta. Brainstem cholinergic systems are also active, and stimulate thalamocortical loops to generate EEG desynchronization similar to wakefulness. During REM sleep, EMG is low, indicating paralysis through motoneuron inhibition (tonic REM sleep). Twitches (bursting of EMG and small movements) also occur, with intermittent saccades of rapid eye movements and pontogeniculooccipital electrical waves (phasic REM sleep). We hypothesize that during REM sleep, basic locomotor, sensory, and thermoregulatory circuits are recovering.

Sleep appears to be both a global phenomenon regulated by sleep regulatory neuronal networks, and a local process within specific brain or cortical areas. Critical brain regions involved in sleep regulation [45–47] are depicted in Figure 1. Early experiments demonstrated the importance of the pons and caudal midbrain for the generation and periodicity of REM sleep [48,49]. For example, periodic atonia and rapid eye movements are still observed downstream after sectioning the pontine/mesencephalic junction, while EEG manifestations of REM sleep are evident after caudal midbrain transections in cats (Figure 1A). A model of mutual inhibition of cholinergic and monoaminergic pontine cell groups [49] was subsequently proposed to regulate REM sleep. In this model, monoaminergic and cholinergic neurons contribute to the EEG desynchronization seen during wakefulness, and they reduce activity during NREM sleep [50–52]. REM sleep is associated with low aminergic tone (e.g., activity), but high cholinergic tone [50–52]. This model is supported by pharmacological studies [53], but has been difficult to substantiate through lesion experiments [45,52,54].

The observation of unihemispheric NREM sleep in some species suggests that sleep can be generated within the cortex and by thalamocortical loops [55]. Imaging studies have shown that deactivation of the thalamus, an integrator of sensory inputs, is a first manifestation of sleep onset [56]. Bilateral lesions of the paramedian thalamus as a result of stroke [57] can lead to profound sleepiness, whereas fatal familial insomnia (a variant of the prion-mediated Creutzfeldt-Jakob disease) involving anterior thalamic nuclei presents with agrypnia [58], a form of insomnia where patients appear sleepy but are unable to fall asleep. Yet large lesions of the thalamus seem to have little effect on the EEG or sleep in animals [59,60], suggesting that other, nonthalamic, lesions may also be involved in stroke or fatal familial insomnia patients.

The importance of the hypothalamus in sleep regulation was suggested by studies of encephalitis lethargica brains during the epidemic of 1918–1924 [61]. Further studies have identified GABAergic sleep-promoting populations in the ventrolateral preoptic (VLPO) [62] and median preoptic [63] regions of the hypothalamus, as well as in the adjacent basal forebrain [64] areas. Other hypothalamic systems, such as the hypocretin/orexin system (which is disrupted in narcolepsy [65]) and the histaminergic tuberomammillary nucleus [66] (located in the posterior hypothalamus), were also discovered. The distribution of these systems—posterior hypothalamus for wake maintenance and anterior hypothalamus-basal forebrain for sleep promotion (and circadian rhythmicity, with the suprachiasmatic nucleus or SCN)—may explain why encephalitis lethargica subjects with posterior and anterior hypothalamic lesions respectively presented with profound sleepiness (as reported in the book Awakenings [67]), or insomnia and sleep inversion [61].

A model building on these findings, where sleep/wake and REM/NREM sleep regulatory populations are mutually inhibitory, creating a series of flip-flop switches, has recently been proposed [62]. The flip-flop is inherently unstable, producing sleep or wake, and ensuring that mixed sleep/wake states do not occur. Systems such as the hypothalamic hypocretin system activate only one side of the switch, ensuring preference for one of these states. The first switch, regulating NREM/wake transitions, includes aminergic and VLPO hypothalamic GABAergic cells. When monoaminergic tone is low, and the organism is profoundly asleep, cholinergic cells of the laterodorsal tegmental and pedunculopontine nuclei in the pons are subsequently disinhibited, activating REM sleep via a second, NREM/REM flip-flop switch. This second switch also includes GABAergic REM-on (sublaterodorsal tegmental nucleus [49] or sublocus coeruleus [45]) and GABAergic REM-off (ventrolateral periaqueductal gray matter and lateral pontine tegmentum) neurons. The REM-on area also contains glutamatergic neurons projecting to the basal forebrain (regulating the EEG) and to the ventromedial medulla and the spinal cord (regulating muscle tone). Strengths of this model, described in Figure 1 with minor modifications, include the emphasis on the need for stability of specific sleep stages and the suggestion that most dysregulations will lead to inherent sleep state instability. A weakness of the model may be the primary use of c-fos to map active regions, and the likelihood that other brain regions of importance in sleep regulation will be discovered.

## The Limitations of Brain Organization Models for Sleep Regulation

Brain localization models are generally insufficient to explain brain function. Only in the unusual case of the SCN has a structure been identified as the major regulator of a function, that is, circadian regulation in mammals [22,62]. In this case, however, it was later shown that many other cells have their own clocks, but that these are only revealed when the master clock is lesioned [62,68,69]. Similarly, specific brain regions such as the hippocampus have been shown to be critical for memory through bilateral lesions, but almost all neuronal networks are able to learn (an example is long-term potentiation [LTP], or increased electrophysiological response after repeated stimulation of a circuit, for example with learning). Likewise, in sleep research, lesion studies or deletion of specific mediators (such as VLPO or hypocretin) can point to the relative importance of some brain regions, but in all cases, sleep/wake still occurs, although occasionally sleep, and often REM sleep, stay diminished [45,52]. It is thus evident that compensation is the rule rather than the exception.

Sleep-state-specific neuronal units can be observed in all parts of the brain [46], and peripheral changes specific to each state also occur [70]. It is thus likely that sleep is a distributed process, but that some neuronal systems (as listed in Figure 1, plus some yet to be identified) are primary drivers. Interactions between sleep- or wake-specific populations of neurons must ensure that the processes occur in synchrony and in exclusion of each other to create stable states of wake, NREM, and REM, with limited time spent in transition states. The delineation of these populations is likely to use mutual inhibition mechanisms as in the flip-flop models described above [45], but probably uses some degree of mutual excitation as well, ensuring that the entire network specific to a state is activated at once [71]. Dysregulation of these systems in human pathologies can lead to sleep/wake instability and state dissociation, as exemplified in narcolepsy, where REM/wake dissociations are frequent, or in parasomnias such as sleep-walking, where NREM/wake dissociation occurs [72].

## Current Theories on Why We Sleep

*Decreased energy demands*: Current theories on why we sleep can be divided into three main groups (Table 1). Based on the observation that long-term sleep deprivation in rats is associated with metabolic dysregulation [14,73], it has been proposed that sleep was selected to reduce energy demands [1,19]. The fact that endothermy and REM/NREM organization are coincidental in both birds and mammals supports this hypothesis. In this model, functioning peaks at specific times in terms of performance (temperature and vision) and food availability (nocturnal, diurnal, crepuscular). It is thus advantageous to reduce energy expenditure at other times, ensuring survival when food is scarce. As sleep is associated with reduced brain energy expenditure, and given that energy consumed by this organ is an increasing fraction of total body energy consumption in organisms with large brains (~30% in humans), sleep may have become more and more important. Models have shown that small energy savings could have effects on selection. Further, sleep amounts, cycle length, and REM sleep time correlate with brain and body size [1].

**Table 1:**
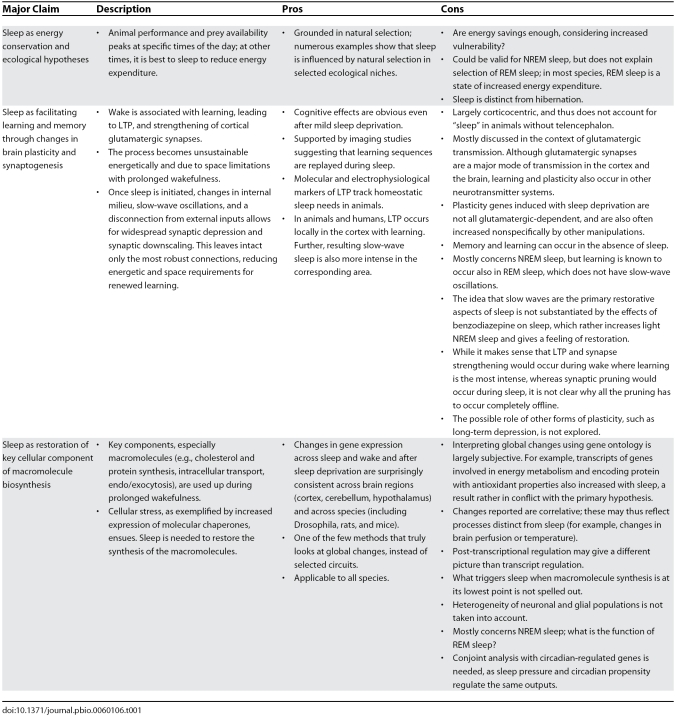
Overview of Common Sleep Theories, Including Their Strengths and Flaws

If sleep was selected to save energy, it should be molecularly linked with this process. A model proposed that adenosine, released as an indicator of low metabolic states by glial cells, could signal increased sleep pressure and initiate sleep [74]. Adenosine release increases in the basal forebrain area after sleep deprivation, and modulates sleep via inhibition of cholinergic basal forebrain neurons [50]. Similarly, metabolic indicators, such as leptin, ghrelin, glucose, adenosine, and adenosine triphosphate, modulate hypocretin neurons. In this case, however, indicators of low metabolic status stimulate hypocretin activity, promoting wake to search for food [75]. Finally, circadian mouse mutants are prone to metabolic abnormalities [76].

Several aspects make the energy economics model insufficient to explain natural selection of sleep. First, if true, sleep would be similar to hibernation, selected to save energy [77]. Against expectation, however, animals coming out of torpor experience a sleep rebound, suggesting sleep deprivation [77]. Further, whereas NREM sleep may be associated with decreased energy expenditure, REM sleep is most often associated with increased whole body oxygen consumption [78,79]. In this latter case, however, it depends on how close ambient temperature is to the thermoneutral range, as REM sleep is a state where temperature is partially unregulated [79]. Overall, whereas it is possible that energy saving has been involved in the selection of sleep at earlier times during evolution or in specific circumstances (for example in mammals with high energy demands, such as mice), it is unlikely to be of primary importance in explaining its maintenance in all mammals.

*Sleep, information processing, and synaptic plasticity*: Higher cortical functions such as cognition, attention, and memory are rapidly affected by sleep deprivation. Studies have shown that learning and memory are improved by subsequent sleep without repetition of the task, suggesting information processing occurs during sleep [80–83]. Further, imaging studies have shown depression in activity in cortical regions involved in a task learned during prior wakefulness during NREM and a reactivation during REM sleep [81,82,84].

Tononi and Cirelli [85,86] proposed that learning during wake leads to LTP and strengthening of glutamatergic synapses, a process that eventually becomes unsustainable (Table 1). Sleep then occurs, leading to a proportional synaptic downscaling that leaves only the most robust connections intact, and reducing energetic and space requirements for the maintenance of crucial learned circuits. Synaptic downscaling would also increase signal-to-noise ratio for remaining connections, improving performance. Accelerated memory transfer from the hippocampus to the cortex could also occur [87].

In support of this hypothesis, phosphorylation of Ser831 AMPA receptors, Thr286 CamKII, and Ser9 GDK3beta occurs in proportion to sleep debt in the cortex and hippocampus, as does increased density of GluR1-containing AMPA receptors [86]. Further, brain-derived neurotrophic factor, Arc, nerve growth factor, alpha subunit, and P-CREB (known to increase with LTP) also increase, as does evidence for LTP after local field stimulation [86]. These effects are independent of light, time of day, and temperature, but track estimated sleep debt. In parallel, the same authors found that slow-wave sleep activity is increased locally [88] and globally by procedures associated with LTP, and decreased with procedures associated with synaptic depression [89]. Interestingly, Rao et al. also found similar molecular changes in the wake-active hypocretin network [90].

The synaptic plasticity model reconciles disparate observations, such as the local occurrence of NREM sleep, temporal association between slow-wave activity and sleep debt, the importance of sleep for learning and memory, and the possibility that sleep is efficient energetically. It does not however consider other forms of learning, such as long-term depression and altered efficacy of inhibitory synapses. Further, the known role of REM sleep in memory consolidation and its association with an activation of hippocampalneolimbic circuits is not considered [91–93]; a differential role of REM versus NREM sleep in consolidating hippocampus-dependent procedural memories versus declarative memories is increasingly suggested [82]. Finally, the model focuses on the telencephalon, and does not discuss how plasticity may occur in sleep-active networks. If the process is general (and extends to species without a telencephalon, such as Drosophila), it is likely to involve other metabolic, structural, or electrophysiological processes, such as replenishment of neurotransmitter stores in terminals through vesicular trafficking. Indeed, for example, administration of neurotransmitter-depleting agents, such as amphetamine, leads to stronger rebounds in sleep time than administration of those preserving dopaminergic storage [94].

*Sleep as restoration of key cellular components of macromolecule biosynthesis*: A large portion of genes in the brain change expression with sleep [95–99], half independently of circadian phase (Table 1). These changes are consistent across species (including Drosophila, rats, mice, and birds) and brain regions (cortex, cerebellum, hypothalamus), and, similar to circadian regulation [100,101], even occur in peripheral organs [102,103]. A large number of sleep-associated transcripts of the brain are involved in glutamatergic transmission, such as homer1a, Arc/Agr3.1, and nptx2 [103], suggesting a link with synaptic plasticity in many glutamatergic synapses. Further, gene ontology analysis found that sleep-associated transcripts encoded proteins involved in the synthesis of complex macromolecular components such as cholesterol and protein synthesis, intracellular transport, and endo/exocytosis [98]. Protein synthesis had been shown to be increased during sleep in older studies [104]. In contrast, wake was associated with the regulation of genes involved in transcription and RNA processing, and at a later stage, with increased expression of molecular chaperones, a finding suggesting cellular stress [98,105]. These results suggest that a function of sleep may be to restore macromolecules [98] and replenish/traffic transmitter vesicles that have been used by extended wakefulness.

A strength of this restoration hypothesis is that it is applicable to all organisms and tissues, as it is cellular-based. It also suggests that sleep is helpful to reverse cellular changes that occur during wakefulness. A major weakness is that changes reported are only correlative, and may not be related to the function of sleep (Table 1).

## Robustness May Favor Temporal Organization of Sleep

Internal stability (homeostasis), whether at the cellular or organismal level, is a prerequisite for life, yet is constantly challenged by external factors. Homeostatic systems must thus be robust and maintain “safety factors”: excess capacity to protect against failure in the face of unpredictable perturbations [106].

Robust homeostasis, once achieved, is difficult to remove [107] and leads to a reduced adaptive potential, as it limits the dynamic range of variation. Additional adaptive mechanisms such as redundancy, modularity, and positive and negative feedback loops [108] are then layered on top of prior homeostatic traits, eventually evolving into an even more robust, more efficient trait [107]. Such a model could explain why sleep (or circadian regulation), once it has evolved, has been a constant phenomenon across evolution. It may also explain why wake- or sleep-promoting neuronal networks (and molecular networks) are layered onto each other, and why discrete brain regions never abolish sleep, as robustness to damage can be achieved through a hierarchical cluster organization with only a few highly connected nodes [109]. The hypocretin system, for example, is a wake-promoting system paradoxically activated by sleep deprivation [110]. In this model, it may be a recent evolutionary addition, increasing (with other systems) the dynamic range of an organism's ability to withstand sleep deprivation (especially in humans, where wakefulness extends through the entire day) [110].

We hypothesize that time organization, in terms of circadian organization (predictive homeostasis), built-in delays (reactive homeostasis), and coordination of various metabolic and cellular processes all act to improve overall robustness in the organism. The rationale for building time delays into sleep recovery may be due to the advantage of recovering when central nervous systems are offline and the possibility of increased efficacy when multiple compatible systems recover in synchrony. At the practical level, recovery is more efficient when a system is inactive (for example, after a physical effort, recovery is easier at rest). In the awake brain, high levels of activity are observed across the entire neuroaxis, reflecting the complexity of active behaviors sustaining reproduction, feeding, and survival. Recovery of wake-active systems in the brain leads to sleep, a state where consciousness is suspended. Further cost savings can be provided by favoring the recovery of compatible molecular pathways, when the organism is least likely to reproduce or find food, for example, during the night in diurnal animals. A similar “intrinsic adaptive value” has been suggested for the coordination of internal metabolic processes by the circadian system [18]. Finally, sleep as a behavior for recovery and return to homeostasis is more flexible than often perceived. Depending on the sleep debt or circadian time, it can be associated with a faster recovery with a more dangerous loss of consciousness (when sleep deprived), or a slower recovery using lighter sleep (and thus a less decreased arousal threshold) [55,111].

## What Remains To Be Solved?

*Homeostatic and circadian regulation: independent or intimately linked?* Circadian and homeostatic regulation of sleep are usually considered distinct [20]. Although this holds true under various experimental conditions, it would be strange if these processes, functionally linked by environmental conditions such as light and dark, had not evolved molecular links. Most recently, it has been reported that sleep deprivation can impair expression of circadian genes [112] and modulate electrical activity within the SCN, a known regulator of circadian rhythms [113]. Further, many circadian mutants [112,114] have abnormal sleep homeostasis, and half of sleep-regulated transcripts are also modulated by circadian time [95,96], suggesting that a simple dichotomy between circadian and sleep homeostasis may not be valid.

*The problem of REM sleep*: Considering the fact that NREM sleep may have localized restorative effects (in particular slow-wave sleep in the cortex), it is tempting to speculate that REM sleep could have a similar role in some noncortical regions. In this case, the REM/NREM duality could have evolved to allow different parts of the brain to come offline. Problematically, however, whereas NREM sleep is associated with decreased metabolic activity and unit firing, REM sleep is an active state with increased energy expenditure [19] and enhanced activity in the pons, amygdala, and most of the cortex [92]. A cessation of neuronal activity during REM sleep is however observed in some key regulatory areas (e.g., monoaminergic cells), and a number of basic homeostatic regulatory processes, such as the regulation of body temperature and various autonomic functions, are offline during REM sleep [70,79]. REM-off neurons are also present in many brainstem regions [115], and are often dismissed as passive monitors of motor activity, as these units often fire during phasic REM sleep. Homeostasis could thus be specifically restored in these networks during REM sleep.

This hypothesis does not explain how REM sleep, a hybrid state with decreased activity in a few networks and increased activity elsewhere, could have evolved. To solve this puzzle, it has been argued that the function of REM sleep has changed across evolution. A primitive state more akin to REM sleep may have emerged first to restore homeostasis in locomotor (many neurons are activity-on or -off in the brainstem), sensory, autonomic, and subsequently thermoregulatory networks [1]. To take thermoregulatory networks offline may have been less costly energetically in animals like reptiles, who have achieved partial endothermy [116]. Such a primitive state may still exist in echidnae, which are partially endothermic mammals [116]. Constant endothermy subsequently evolved, favoring continuing activity in the cold and dark of temperate climates and also rendering sleep more and more costly energetically. Sleep would have then diverged into two states: NREM sleep to restore metabolic homeostasis in most of the brain, and REM sleep to restore selected primitive networks mentioned above. Activation of forebrain and limbic areas during REM sleep [92] would have finally been selected to optimize learning and creativity, increasing survival and mitigating the negative effects of increased energy expenditure. Indeed, similar to NREM sleep, functional imaging studies have shown “replay” of neuronal activation sequences that have been learned during the prior day during REM sleep [51,84]. Further, REM sleep deprivation has strong effects on memory consolidation [80,81]. This hypothesis may also explain why long-term REM sleep deprivation is lethal, as it would also perturb primitive networks involved in energy homeostasis and basic functions.

The fact that REM sleep is not easily observed in some rare mammals may only reflect difficulties in measuring this process in the right networks, variations in forebrain activation, and strong effects of natural selection in selected ecological instances. It may also explain the complex phenotype of REM sleep (erections, atonic and phasic motor activity, and rapid eye movement), as it is possible that REM sleep is ancestrally the summation of several distinct substates. These speculations strongly argue for the need to study molecular and electrophysiological changes within important structures (e.g., cortex) and discrete sleep and wake regulatory networks, starting with the best characterized (Figure 1), rather than globally. We predict for example that recovery in sleep-promoting networks will occur during wake, with a similar molecular signature (but allowing for neurochemical diversity, as many of the wake-active systems currently reported are glutamatergic or monoaminergic while most known sleep-promoting systems are GABAergic; see Figure 1).

*Molecular and anatomical studies of sleep and sleep regulatory networks across species*: To conduct molecular studies in exotic species is increasingly easy, thanks to genomic sequencing efforts, yet there are almost no data on the functional organization of sleep regulatory networks across species. Recent studies have shown that hypocretin, a major regulator of monoaminergic tone and sleep in mammals, does not have similar anatomic connections, has only one rather than two receptors, and is not strongly wake-promoting in fish [32], where light and melatonin have more effects [29,31,32]. Similarly, birds, which are very sensitive to light and melatonin, also have a single hypocretin receptor, as does the marsupial opossum, an animal with large amounts of REM sleep. This suggests that the top neural networks orchestrating the occurrence of sleep are more variable across species than are cellular, molecularly based changes; this is analogous to the circadian system, where clock genes are more conserved than SCN organization [117]. Further studies in selected species will be extremely instructive in understanding sleep across evolution, confirming or rejecting some of the hypotheses discussed above.

## Conclusion

Sleep is as necessary as water and food, yet it is unclear why it is required and maintained by evolution. Recent work suggests multiple roles, a correlation with synaptic plasticity changes in the brain, and widespread changes in gene expression, not unlike what has been recently discovered in circadian biology. Functional data are however still largely lacking, and studies such as functional genomic screens in model organisms, comparative sleep neuroanatomy through phylogeny, and the study of molecular changes within specific wake, REM sleep, and NREM sleep regulatory systems are needed. The resilience of behavioral sleep in evolution and after experimental manipulations may be secondary to the fact that it is grounded at the molecular, cellular, and network levels.

## Abbreviations

CREB: cyclic AMP response element-binding protein
EEG: electroencephalogram
GABA: gamma-aminobutyric acid
LTP: long-term potentiation
NREM: non-rapid eye movement
REM: rapid eye movement
SCN: suprachiasmatic nucleus
VLPO: ventrolateral preoptic
